# Aluminum electrolytes for Al dual-ion batteries

**DOI:** 10.1038/s42004-020-00365-2

**Published:** 2020-08-28

**Authors:** Kostiantyn V. Kravchyk, Maksym V. Kovalenko

**Affiliations:** 1 Laboratory for Thin Films and Photovoltaics, Empa – Swiss Federal Laboratories for Materials Science and Technology, Überlandstrasse 129, CH-8600 Dübendorf, Switzerland; 2 Laboratory of Inorganic Chemistry, Department of Chemistry and Applied Biosciences, ETH Zürich, Vladimir-Prelog-Weg 1, CH-8093 Zürich, Switzerland

**Keywords:** Corrosion, Energy, Solid-state chemistry, Batteries

## Abstract

In the search for sustainable energy storage systems, aluminum dual-ion batteries have recently attracted considerable attention due to their low cost, safety, high energy density (up to 70 kWh kg^−1^), energy efficiency (80–90%) and long cycling life (thousands of cycles and potentially more), which are needed attributes for grid-level stationary energy storage. Overall, such batteries are composed of aluminum foil as the anode and various types of carbonaceous and organic substances as the cathode, which are immersed in an aluminum electrolyte that supports efficient and dendrite-free aluminum electroplating/stripping upon cycling. Here, we review current research pursuits and present the limitations of aluminum electrolytes for aluminum dual-ion batteries. Particular emphasis is given to the aluminum plating/stripping mechanism in aluminum electrolytes, and its contribution to the total charge storage electrolyte capacity. To this end, we survey the prospects of these stationary storage systems, emphasizing the practical hurdles of aluminum electrolytes that remain to be addressed.

## Abstract (web summary)

Aluminum dual-ion batteries have attracted considerable attention due to their low cost, safety, high energy density, energy efficiency, and long cycling life. Here the authors review working principles, electrolytes, and corrosion effects of this battery type.

## Introduction

The integration of intermittent renewables into the grid is directly linked to the deployment of stationary energy storage systems at the terawatt scale, enabling grid stabilization. From this perspective, in addition to conventional energy storage means, such as pumped-storage hydroelectricity (PSH), stationary batteries will be of significant importance^1^. Loosely speaking, the assessment of the battery technologies for stationary storage applications can be made by comparing their capital cost (¢ kW^−1^ h^−1^ cycle^−1^) to that of PSH, which is presently the predominant stationary storage system. Consequently, stationary batteries should possess an exceptional cycling stability (thousands of cycles), environmental friendliness, low CO_2_ footprint, and low cost. In this framework, the exploration of batteries composed of Na^2,3^, K^4^, Mg^5,6^, and Al^7–9^ as earth-abundant metals has become a primary research target in recent years. Notably, batteries that employ Al metal as an anode can harness numerous advantages, such as a high charge storage capacity of 2977 mAh g^−1^ of Al, its natural abundance, and safety^10–15^. Furthermore, Al can be reversibly deposited and stripped in chloroaluminate ionic liquids with a high coulombic efficiency and without the formation of dendrites^16,17^. In this context, a new electrochemical concept called the aluminum dual-ion battery (ADIB) has recently attracted significant attention. ADIBs have a high potential for grid-scale energy storage applications owing to their low cost, relatively high energy densities of up to ≈70 Wh kg^−1^^18^, and cyclic stability. In this review, we discuss recent developments in Al electrolytes for ADIBs covering the topics of charge storage capacity and the operating mechanism of ADIBs. In addition, we analyze in detail the impacts of acidity, ionic conductivity, the Al^3+^/Al redox potential, and the electrochemical voltage window of the Al electrolytes on the performance of ADIBs. Finally, with respect to the practical application of ADIBs, the compatibility of current collectors with Al electrolytes is covered in the last section of this review.

## Historical aspects of the development of Al electrolytes for ADIBs

**Fig. 1:**
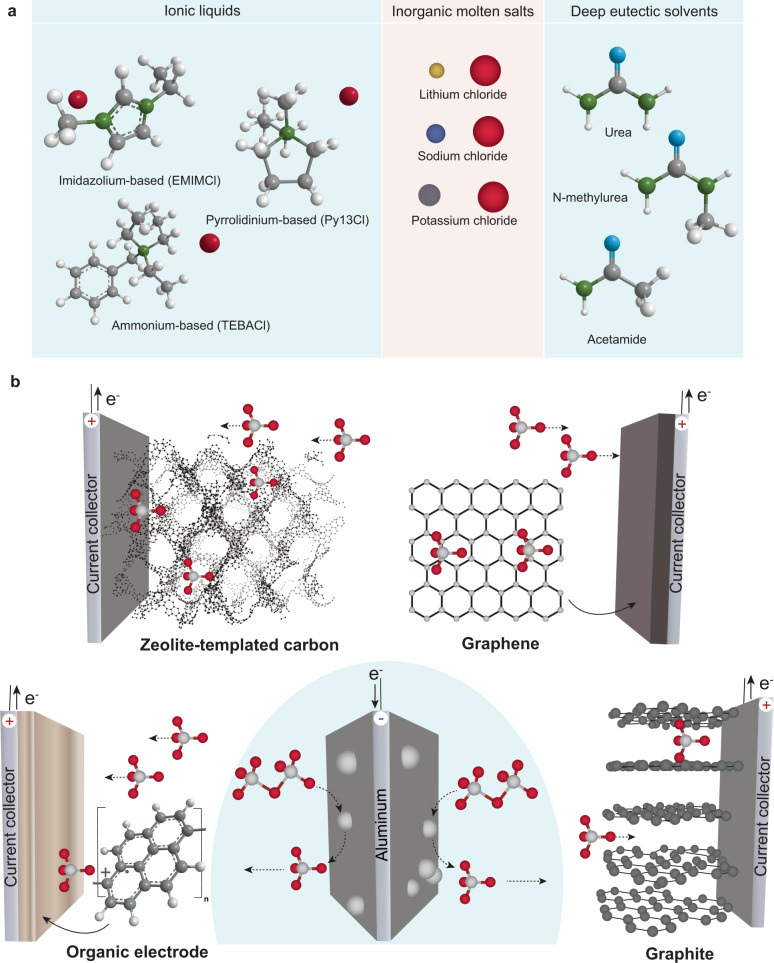
Schematics of different classes of Al electrolytes and working principle of ADIBs. **a** Schematic illustration of the different classes of Lewis bases used for the preparation of Al electrolytes in ADIBs. Chlorine, carbon, hydrogen, and nitrogen atoms are shown in red, gray, white, and green colors, respectively. **b** Schematic of the charging process of ADIBs composed of aluminum foil at the negative electrode, graphite/graphene/zeolite-templated carbon/polypyrene at the positive electrode, and a chloroaluminate ionic liquid electrolyte. Chlorine and aluminum atoms are shown in red and gray colors, respectively.

Although the research on Al electrolytes for ADIBs may appear to be a new subject, this is a misconception. The employment of Al electrolytes in ADIBs based on molten salts was assessed for the first time in the 1970s by Fouletier et al.^19^ Fouletier’s ADIB was composed of metallic aluminum and graphite as negative and positive electrodes, respectively, which were immersed into molten LiCl/AlCl_3_ salts (ca. 140 °C). In 1988, Gifford et al.^20^ extended research on ADIBs towards the use of room-temperature ionic liquids (RTILs) based on imidazolium chemistry (AlCl_3_:1,2-dimethyl-3-propylimidazolium chloride). In 2015, Al electrolytes received a great deal of attention since the publication of Dai et al.^21^ on ADIBs employing a metallic Al anode, two synthetic forms of graphite as the cathode (CVD-grown graphitic foam and pyrolytic graphite) and 1-ethyl-methylimidazolium ionic liquid electrolyte (AlCl_3_:EMIMCl)^21^. Dai’s ADIB showed a high reversibility over thousands of cycles and graphite cathodic capacities of up to 67 mAh g^–1^. Those first publications have initiated the exploration of deep eutectic solvents (DESs) as Al electrolytes for ADIBs by Dai et al.^22,23^, Jiao et al.^24–26^, and others^27–31^. A schematic illustration of the different classes of Al electrolytes presently used in ADIBs is shown in Fig. 1a. A comparison of the various Al electrolytes with respect to their density, viscosity, ionic conductivity, theoretical charge storage capacity, and cost is given in Table 1.

**Table 1:** Comparison of various Al electrolytes for ADIBs with respect to their density, viscosity, ionic conductivity, theoretical charge storage capacity, and cost.

Aluminum electrolyte	ρ (g ml^−1^)	η (cP)	σ (mS cm^−1^)	Theoretical capacity (mAh g^−1^)	Cost (USD/kg^−1^)
AlCl_3_/LiCl/KCl^84^ (1.44/0.71/0.29)	–	–	–	36.28	13.4^66^
AlCl_3_/LiCl/KCl^66^ (1.4/0.64/0.36)	–	–	160^66^ (100 °C)	33.41	–
AlCl_3_/NaCl/KCl^66^ (1.52/0.64/0.36)	–	–	190^66^ (100 °C)	39.158	–
AlCl_3_/NaCl/LiCl^66^ (1.31/0.5/0.5)	–	–	170^66^ (100 °C)	27.68	–
AlCl_3_/NaCl/LiCl/KCl^66^ (1.31/0.43/0.42/0.15)	–	–	170^66^ (100 °C)	27.82	12^66^
AlCl_3_:NaCl^85^ (1.63:1)	–	–	190^85^ (120 °C)	45.92	9.5^66^
AlCl_3_:NaCl^65^ (2:1)	–	–	–	61.82	9.7^66^
AlCl_3_:EMIMCl^15, 18, 21, 32, 33, 35, 47, 62, 86, 87^					
*r* = 1.0	1.295^86^	–	–	0	–
*r* = 1.1	1.308^86^	–	–	6.85	–
*r* = 1.2	1.318^86^	–	–	13.11	–
*r* = 1.3	1.332^86^	–	14.2^47^	18.85	71.3^66^
*r* = 1.4	1.341^86^	16.34^86^	17.43^86^	24.12	–
*r* = 1.5	1.354^86^	15.60^86^	17.03^86^	29	66.8^66^
*r* = 1.6	1.365^86^	16.04^86^	15.00^86^	33.5	–
*r* = 1.7	1.374^86^	15.16^86^	16.06^86^	37.69	–
AlCl_3_:PMIMCl^47^					
*r* = 1.3	–	–	12.8^47^	18.08	–
AlCl_3_:PDMIMCl^47^					
*r* = 1.3	–	–	5.3^47^	17.84	–
AlCl_3_:BDMIMCl^47^					
*r* = 1.3	–	–	2.7^47^	17.14	–
AlCl_3_:BMIMCl^47, 60^					
*r* = 1.3	–	–	9.2^47^	17.33	–
AlCl_3_:TEBAC^48^					
*r* = 2.2	–	–	0.454^48^	46.29	–
*r* = 2.3	–	–	0.520^48^	48.89	–
*r* = 2.4	–	–	0.539^48^	51.37	–
*r* = 2.5	–	–	0.501^48^	53.73	–
AlCl_3_: Py13Cl^86^					
*r* = 1.4	1.303^86^	56.58^86^	5.15^86^	22.95	–
*r* = 1.5	1.276^86^	45.23^86^	5.15^86^	27.63	–
*r* = 1.6	1.305^86^	38.30^86^	5.11^86^	31.99	–
*r* = 1.7	1.334^86^	41.99^86^	4.84^86^	36.04	–
AlCl_3_/ImidazoleHCl^88^					
*r* = 1.4	–	–	0.237^88^	27.61	–
*r* = 1.5	–	–	0.279^88^	32.99	–
*r* = 1.6	–	–	0.256^88^	37.94	–
AlCl_3_/Et_3_NHCl^29–31^					
*r* = 1.4	1.265^29^	31.8^29^	8.86^29^	–	–
*r* = 1.6	1.273^29^	29.8^29^	8.35^29^	–	–
*r* = 2.0	1.312^29^	25.3^29^	7.69^29^	–	–
AlCl_3_:urea^22, 23^					
*r* = 1.0	1.56^22^	133.2^22^	1.02^22^	–	–
*r* = 1.2	1.59^22^	113.8^22^	1.10^22^	–	10^66^
*r* = 1.4	1.6^22^	87.1^22^	1.12^22^	21.35	10^66^
*r* = 1.5	1.61^22^	88.9^22^	1.17^22^	–	10^66^
AlCl_3_ is not soluble at *r* > 1.5^22^					
AlCl_3_: N-methyl-urea^22^					
*r* = 1.0	1.46^22^	86.6^22^	1.19^22^	–	–
*r* = 1.2	1.5^22^	87.6^22^	1.18^22^	–	–
*r* = 1.4	1.51^22^	67.2^22^	1.27^22^	15.97	–
*r* = 1.5	1.52^22^	77.7^22^	1.21^22^	–	–
AlCl_3_ is not soluble at *r* > 1.6^22^					
AlCl_3_: N-ethyl-urea^22^					
*r* = 1.0	1.38^22^	52.4^22^	1.43^22^	–	–
*r* = 1.2	1.41^22^	48.0^22^	1.52^22^	–	–
*r* = 1.4	1.43^22^	45.0^22^	1.56^22^	18.27	–
*r* = 1.5	1.45^22^	44.7^22^	1.49^22^	–	–
AlCl_3_ is not soluble at *r* > 1.5^22^					

The theoretical capacities of RTILs and inorganic molten salts were calculated using expression Eq. 3. The theoretical capacities of deep eutectic solvents were computed from Eq. 5 (see Supplementary Note 1) using the concentration of Al_2_Cl_7_^−^ ions in AlCl_3_:urea, AlCl_3_:Me-urea and AlCl_3_:Et-urea electrolytes reported in the ref. ^22^.

## Working principle of ADIBs and Al electrolytes based on RTILs

The basic configuration of ADIBs comprises a carbonaceous (graphite, zeolite-templated carbon (ZTC), or graphene) or organic positive electrode, chloroaluminate ionic liquid electrolyte and metallic aluminum anode as demonstrated in Fig. 1b. ADIBs operate as an electrochemical energy storage system employing reversible intercalation/insertion of the AlCl_4_^−^ anion species into the positive electrode upon charge (oxidation). Concomitantly, the electroplating of aluminum occurs at the negative electrode of ADIBs. The working principle of ADIBs can be represented by the following cathodic and anodic half-reactions during charge:

1 $${\mathrm{On}}\,{\mathrm{the}}\,{\mathrm{negative}}\,{\mathrm{electrode}}:4{\rm{Al}}_2{\rm{Cl}}_7^ - + 3{\rm{e}}^ - \leftrightarrow 7{\rm{Al}}{\rm{Cl}}_4^ - + {\rm{Al}},$$

2 $${\mathrm{On}}\,{\mathrm{the}}\,{\mathrm{positive}}\,{\mathrm{electrode}}:x{\rm{C}} + {\rm{Al}}{\rm{Cl}}_4^ - \leftrightarrow {\rm{C}}_x\left( {{\rm{Al}}{\rm{Cl}}_4^ - } \right) + {\rm{e}}^ -,$$

where C is graphite^32^, ZTC^33^, graphene^34^ or organic active material^35,36^. Consequently, the mechanism of ADIBs is significantly different from the “rocking-chair” metal-ion batteries. There is no one-directional motion of Al^3+^ ions from the positive to the negative electrodes. Al species are depleted from the chloroaluminate ionic liquid during the charge of ADIBs and are consumed by both electrodes. The Al electrolytes that are used in ADIBs play a double function: they support the Al plating/stripping process and act as the source of AlCl_4_^−^ ions being needed for the intercalation/insertion into the positive electrode during charge. Consequently, ADIB term is applied for batteries, were electrolyte acts as a source of two types of ions (i.e., Al_2_Cl_7_^−^ and AlCl_4_^−^) required for electrochemical energy storage. Thus, the composition of the electrolyte is changing upon charge and discharge, contrary to “rocking-chair” Al-ion battery systems, where electrolyte acts exclusively as a transmitter of the ions. The most conventional example of an Al electrolyte is a mixture of aluminum chloride and other chlorides comprising an organic cation, for instance, 1-butyl-3-methylimidazolium chloride (BMIM) and 1-ethyl-3-methylimidazolium chloride (EMIM). As a consequence of the acid–base interactions between AlCl_3_ (Lewis acid) and Cl^-^ (Lewis base), the salt mixture becomes a liquid at room temperature, forming an RTIL. The latter is composed of AlCl_4_^−^ anions that are charge-balanced with organic cations. The RTIL with an excess of Lewis acid AlCl_3_ over Lewis base EMIMCl is comprised of both AlCl_4_^−^ and Al_2_Cl_7_^−^ ions. Importantly, ADIBs are operational only in acidic formulations. Solely Al_2_Cl_7_^−^ ions enable the electroplating of aluminum, which therefore, occurs only in chloroaluminate melts with an excess of AlCl_3_^37–46^. As a result, the charge storage capacity of the chloroaluminate melt is a function of the concentration of Al_2_Cl_7_^−^ ions in the RTIL. Electroplating, and therefore, the charging process stops when no Al_2_Cl_7_^−^ ions are left in the ionic liquid, which results in the formation of the neutral melt (AlCl_3_:EMIMCl = 1). The highest molar ratio (*r*) between AlCl_3_ and EMIMCl that forms an RTIL is ca. 2:1. AlCl_3_ does not dissolve at higher molar ratios. Apart from the ionic melts based on AlCl_3_/EMIMCl, other RTILs have also been recently reported to be composed of 1-methyl-3-propylimidazolium chloride (MPIMCl)^47^, benzyltriethylammonium chloride (TEBACl)^48^, and 1,2-dimethyl-3-propylimidazolium chloroaluminate (DMPIMCl)^49^.

**Fig. 2:**
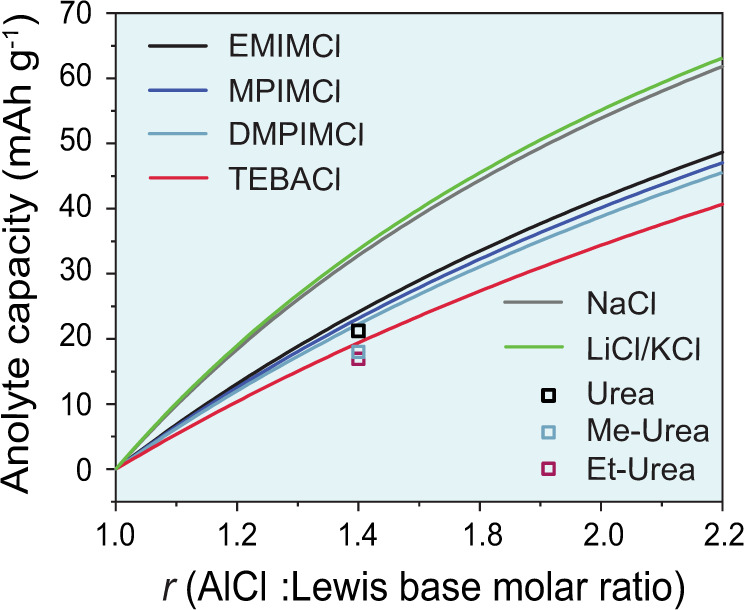
The charge storage capacity of the RTILs, inorganic molten salts and deep eutectic solvents versus their acidity (*r*). RTILs: AlCl_3_:1-ethyl-3-methylimidazolium chloride (EMIMCl), AlCl_3_:1-methyl-3-propylimidazolium chloride (MPIMCl), AlCl_3_:1,2-dimethyl-3-propylimidazolium chloroaluminate (DMPIMCl) and AlCl_3_:benzyltriethylammonium chloride (TEBACl). The curves for RTILs and inorganic molten salts are computed from Eq. 3. The points for deep eutectic solvents are computed from Eq. 5 (see Supplementary Note 1) using the concentration of Al_2_Cl_7_^−^ ions in AlCl_3_:urea, AlCl_3_:Me-urea and AlCl_3_:Et-urea electrolytes reported in the ref. ^22^.

Figure 2 illustrates the impact of acidity *r* on the charge storage capacity of the chloroaluminate ionic liquid electrolytes, which can be, in fact, called anolytes. The theoretical gravimetric and volumetric capacities of the ionic liquid *C*_an_ can be calculated as follows:

3 $${\mathrm{Gravimetric}}\,C_{{\rm{an}}} = \frac{{Fx\left( {r - 1} \right)}}{{rM_{{\rm{AlCl}}_3} + M_{{\rm{ACl}}}}}({\mathrm{mAh}}\,{\mathrm{g}}^{ - 1}),$$

4 $${\mathrm{Volumetric}}\,C_{{\rm{an}}} = \frac{{Fx\left( {r - 1} \right)\rho }}{{rM_{{\rm{AlCl}}_3} + M_{{\rm{ACl}}}}}\,({\mathrm{mAh}}\,{\mathrm{g}}^{ - 1}),$$

where *F* = 26.8 × 10^3^ mAh mol^−1^ (the Faraday constant), $x = \frac{3}{4}$ (number of electrons that are used to reduce 1 mol of the Al_2_Cl_7_^−^ ions), *M*_AlCl3_ is the molar mass of AlCl_3_ (g mol^−1^), *M*_ACl_ is the molar mass of the Cl^−^ salt (g mol^−1^), *r* is the AlCl_3_:ACl molar ratio, and *ρ* is density of the chloroaluminate melt (g mL^−1^). A detailed description of the derivation of Eqs. 3 and 4 can be found in ref. ^50^.

**Fig. 3:**
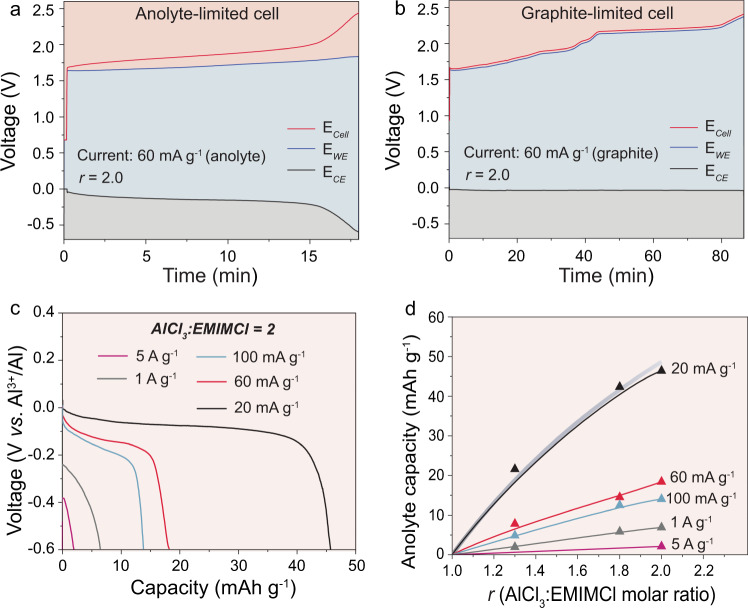
Electrochemical performance of AlCl3:EMIMCl chloroaluminate ionic liquid. **a**, **b** Galvanostatic voltage profiles for the AlCl_3_:EMIMCl chloroaluminate ionic liquid (*E*_CE_, negative electrode), graphite (*E*_WE_, positive electrode), and full cell (*E*_Cell_) vs. the Al reference electrode in anolyte-limited (**a**) and graphite-limited (**b**) cell configurations. **c** Galvanostatic discharge curves of the AlCl_3_:EMIMCl anolyte (negative electrode) measured in the anolyte-limited cell configuration at different currents in combination with graphite and aluminum as the working (positive) and the reference electrodes, respectively. **d** Specific gravimetric capacities of the AlCl_3_:EMIMCl anolyte with *r* = 1.3, 1.8, and 2.0 measured at different currents. The gray line shows the theoretical capacity of the AlCl_3_:EMIMCl anolyte computed from Eq. 3. Adapted from ref. ^58^, ACS.

For instance, the gravimetric charge storage capacities of the AlCl_3_:EMIMCl ionic liquid are equal to 19 mAh g^−1^ and 48 mAh g^−1^ for *r* = 1.3 and *r* = 2, accordingly. Notably, these capacities define the overall energy density of ADIBs^14,18,50–57^. Moreover, it should be pointed out that these theoretical capacities are not always achievable experimentally, i.e., they depend on practically relevant experimental conditions and on whether Al_2_Cl_7_^−^ ions can be fully depleted for Al electroplating. To figure out this point, recently, Kravchyk et al.^58^ assembled the anolyte-limited cell in a three-electrode configuration with a significant excess of graphite cathode (anolyte-limited cell). Upon these measurements (Fig. 3a), in addition to the voltage profile of the full cell, the profiles for both positive and negative electrodes were recorded. It was demonstrated that the voltage profile at the negative electrode (*E*_CE_) remained relatively stable during charging for 15 min. Upon further charging, however, the voltage dropped sharply indicating the end of the Al plating process caused by the depletion of Al_2_Cl_7_^−^ ions at the negative electrode. Importantly, the voltage profile at the negative electrode (*E*_CE_) for the graphite-limited cell was constant during the entire charge, with a small overpotential of <50 mV (Fig. 3b) pointing to the access of Al_2_Cl_7_^−^ ions at the negative electrode. Using this approach, Kravchyk et al.^58^ performed rate capability measurements of anolyte-limited full cells at different current densities ranging from 5 to 20 mA g^−1^ for the AlCl_3_:EMIMCl ionic liquid formulations with *r* = 1.3–2.0 (see Fig. 3c, d). These experiments revealed two main points. First, as expected, the higher capacities of chloroaluminate melts could be obtained only using highly acidic formulations. For instance, the charge storage capacity of the AlCl_3_:EMIMCl anolyte with *r* = 1.3 was measured to be ca. 21 mAh g^−1^ at a current density of 20 mA g^−1^. In contrast, at *r* = 2, the capacity was ca. 46 mAh g^−1^. These results indicate that the highest energy density of the ADIBs can be obtained using chloroaluminate ionic liquids with *r* = 2, and, therefore, future works on ADIBs should be focused on the most acidic formulations. Second, the applied current density affects the charge storage capacity of the anolyte. This is reflected in the significant deviation in the voltage profiles of the negative electrode at high currents (Fig. 3a). As a result, low charge storage capacities (ca. 10–14% from theoretical values) were obtained at very high current densities of 1 A g^−1^. These results suggest that the frequent statements regarding the high power density of ADIBs are not fully correct. Specifically, at high current densities, a significant drop in the energy density of ADIBs is foreseen. The latter is caused by the rate capability limitations of both the chloroaluminate ionic liquid anolytes and the cathodes of ADIB. In fact, these observations show that the charge storage capacities of the anolyte significantly deviate from the theoretical value at charge current densities higher than 20 mA g^−1^.

Apart from the charge storage capacity, the acidity of the chloroaluminate ionic liquid electrolytes strongly influences their ionic conductivity. For instance, as revealed by Ferrera et al.^59^ for the AlCl_3_–EMIMCl chloroaluminate ionic liquid, its conductivity gradually decreases from 20 to 10 mS cm^−1^ at room temperature upon increasing the AlCl_3_/EMIMCl molar ratio from 1.1 to 1.7, respectively. Furthermore, it was shown by Wang et al.^60^, that the ionic conductivity increases with decreasing halide ionic radius (from iodide to bromide and chloride anions). Moreover, the acidity variations strongly influence the electrochemical stability window of the electrolyte. Specifically, it has been determined that the oxidation of the AlCl_3_: 1-ethyl-buthyl-imidazolium ionic liquid with mole ratios of 1, 1.1, 1.5 and 2 takes place at approximately 2.6 V vs. Al^3+^/Al, while for the 0.8 mole ratio, the oxidation starts at 1.75 V vs. Al^3+^/Al. The oxidation stability limit is associated with the chlorine evolution at the cathode side^41,61^. It should be noted that Wang et al.^62^ assessed the Al^3+^/Al redox potential in the AlCl_3_–EMIMCl ionic liquid vs. the standard hydrogen electrode (SHE) using a specially made cell composed of a β-alumina solid-state electrolyte and Na reference electrode. It was stated that plating/stripping of aluminum in AlCl_3_–EMIMCl ionic liquid occurs at −0.7 V vs. SHE and 2.3 V vs. Li^+^/Li. This Al^3+^/Al redox potential is ca. 1 V higher than that observed in aqueous solutions.

## Inorganic molten salts

Another type of electrolyte capable of reversible aluminum plating/stripping electrochemistry are low-cost molten salt eutectics. One of the first investigations of aluminum electrodeposition in molten salt electrolytes was undertaken by Del Duca in 1971^63^. This work on AlCl_3_–NaCl and AlCl_3_–(LiCl–KCl) mixtures elucidated the kinetics of aluminum electroplating. Gale et al.^64^ examined the subvalent ion effect during aluminum anodization in molten AlCl_3_–NaCl. The melting point of a chloroaluminate eutectic mixture is dependent on its precursors and composition. The typical binary AlCl_3_/NaCl and AlCl_3_/KCl systems melt at above 108 and 128 °C, respectively^65^. The addition of a third/fourth salt to the binary electrolyte can further reduce the melting point. For example, a ternary AlCl_3_/NaCl/KCl electrolyte (molar ratio: 61/26/13) has been reported to melt below 100 °C^65^, and the AlCl_3_/LiCl/KCl system with a molar ratio of 59/29/12 turns entirely liquid at approximately 95 °C. Recently, the quaternary AlCl_3_/NaCl/LiCl/KCl inorganic molten salt with the lowest eutectic temperature of less than 75 °C was also systematically studied as the electrolyte in an ADIB^66^. Apart from the low cost, among the advantages of inorganic molten salts as electrolytes for ADIBs are their low viscosity and high ionic conductivity that facilitate the kinetics of AlCl_4_^−^ insertion/deinsertion reactions. Additionally, with respect to the charge storage capacity, the lower molar mass of alkali chlorides yields higher values of gravimetric capacity over imidazolium or pyridinium chlorides at the same acidity (Fig. 2).

## Deep eutectic solvents

In the search for a more economical and environmentally sustainable alternative to imidazolium-based RTILs, a new class of ionic liquids, namely, DESs, were recently employed as Al electrolytes for ADIBs^43,44,67^. They are also known as ionic liquid analogs (ILAs)^67^. DESs can be defined as a mixture of a strongly Lewis acidic metal halide and an oxygen donor amide ligand, such as urea, acting as a Lewis base. Similar to the AlCl_3_–EMIMCl system^41^, AlCl_3_–urea forms through the exothermic reaction between AlCl_3_ and urea according to the following equation:

5 $$2{\rm{AlCl}}_3 + 2\,{\rm{urea}} \to {\rm{AlCl}}_4^ - + [{\rm{AlCl}}_2\left( {{\rm{urea}}} \right)_2]^ +.$$

**Fig. 4:**
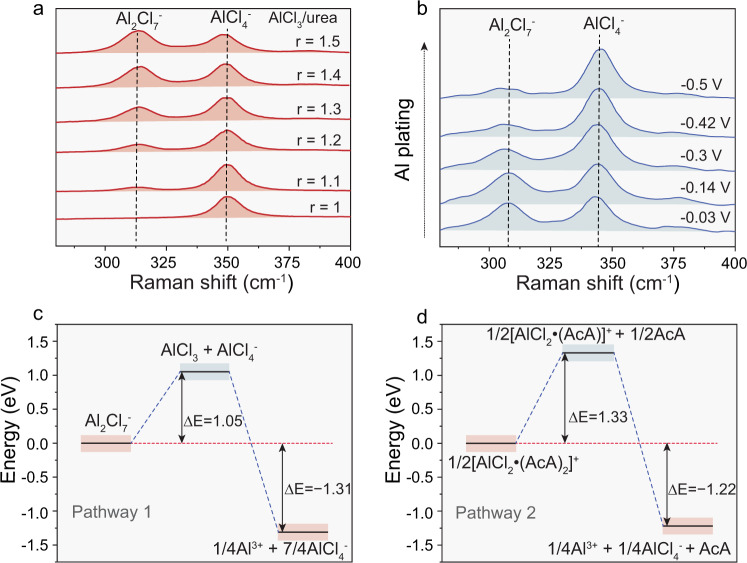
Raman spectroscopy measurements and Al plating pathways of deep eutectic solvents. **a** Raman spectra of the AlCl_3_/urea anolyte (*r* = 1.0, 1.1, 1.3, 1.4, and 1.5); Adapted from ref. ^27^, Elsevier. **b** Operando Raman spectroscopy of the AlCl_3_/urea anolyte (*r* = 1.4) during Al plating at different voltages vs. Al^3+^/Al. The Raman spectra are normalized to the urea C–N symmetric stretch peak at 1050 cm^−1^. Adapted from ref. ^22^, Wiley. **c**, **d** Energy profiles of the dissociation reactions of Al_2_Cl_7_^−^ (c) and [AlCl_2_·(AcA)_2_]^+^ (d). Adapted from ref. ^70^, Elsevier.

The comprehensive characterization of DESs by both Raman^22,24,27,68^ and NMR^22,23,68^ spectroscopies have revealed that only AlCl_4_^−^ are present in the AlCl_3_–urea DESs at an AlCl_3_/urea ratio of 1.0 (neutral). However, as the acidity of the melt increases through the addition of AlCl_3_, the concentration of the Al_2_Cl_7_^−^ species gradually increases when compared to the concentration of the AlCl_4_^−^ species (Fig. 4a). The highest molar ratio (*r*) between the AlCl_3_ and urea that forms an ionic liquid is ca. 1.5:1^22^. Importantly, as revealed by Ng et al.^27^, Al electroplating takes place only in the acidic melts (AlCl_3_/urea > 1.1). Similar observations have also been reported in other AlCl_3_–amide systems^69^. Aiming to identify which species are responsible for Al electrodeposition, Dai et al.^22^ performed *operando* Raman spectroscopy measurements of AlCl_3_–urea DESs (AlCl_3_/urea = 1.4) during Al deposition. Upon Al plating (0 to −0.5 V vs. Al^3+^/Al), a large decrease in the intensity of the Al_2_Cl_7_^−^ peak (313 cm^−1^) and a corresponding increase in the AlCl_4_^−^ peaks (350, 445 cm^−1^) were observed (Fig. 4b). Upon Al stripping (0–0.5 V vs. Al^3+^/Al), the exact opposite processes were observed, namely, there was a large intensity increase and decrease in the Al_2_Cl_7_^−^ and the AlCl_4_^−^ peaks, respectively; no changes to any other peaks were detected. According to Dai’s observations, the Al electrodeposition/stripping processes in AlCl_3_–urea DESs can be described as follows: $4{\rm{Al}}_2{\rm{Cl}}_7^ - + 3{\rm{e}}^ - \leftrightarrow 7{\rm{Al}}{\rm{Cl}}_4^ - + {\rm{Al}}$. We note that previously^23,26^, it had been assumed that the cationic aluminum species $[{\rm{Al}}{\rm{Cl}}_2\left( {{\rm{area}}} \right)_2]^ +$ were responsible for the Al electroplating reaction. This conclusion is in line with the work of Chu et al.^70^, whereby the Al plating pathways were investigated by density functional theory (DFT) calculations (Fig. 4c, d). Considering the difference in the dissociation energy barriers of Al_2_Cl_7_^-^ and $[{\rm{Al}}{\rm{Cl}}_2\left( {{\rm{urea}}} \right)_2]^ +$ is 0.28 eV, it has been assumed that the dissociation of $[{\rm{Al}}{\rm{Cl}}_2\left( {{\rm{urea}}} \right)_2]^ +$ is less kinetically preferred, therefore, favoring the Al plating through the Al_2_Cl_7_^-^ reduction. From this perspective, taking into consideration the concentration of Al_2_Cl_7_^−^ in the acidic AlCl_3_–urea DESs, the charge storage capacity equals ca. 21 mAh kg^−1^ (for AlCl_3_/urea = 1.4). This value can be derived from Eq. 5 (see Supplementary Note 1) using the concentration of Al_2_Cl_7_^−^ ions in AlCl_3_:Urea DES reported in the ref. ^22^. For AlCl_3_:Me–urea and AlCl_3_:Et-urea anolytes, one can obtain similar capacities of ca. 16 mAh g^−1^ and 18 mAh g^−1^, respectively.

In addition to AlCl_3_–urea DESs, several articles have been published reporting the ability of acetamide (AcAm) to form eutectic mixtures when mixed with AlCl_3_, yielding the reversible electrodeposition of aluminum^28,71^. As stated in ref. ^28^, the AlCl_3_–AcAm DES comprises the anionic AlCl_4_^−^ and Al_2_Cl_7_^−^ species as well as complexed aluminum cationic species, such as [AlCl_2_(AcAm)_2_]^+^. Another eutectic AlCl_3_–organic solvent system with a coordination behavior similar to that of AlCl_3_–amide is the AlCl_3_–diglyme electrolyte, which also supports reversible Al deposition/dissolution^72,73^. The active species in this electrolyte are AlCl_2_(diglyme)_2_^+^ cations, which are paired with the AlCl_4_^−^ anions.

Considering the low cost of DESs and their environmental friendliness, energy storage systems that utilize DES electrolytes will have significant economic and environmental cost advantages over conventional RTILs. However, such systems have a relatively low electrochemical stability window of 2.2 V vs. Al^3+^/Al in comparison with that of dialkylimidazolium chloride-based RTILs^22,23,27^. The latter are stable up to ∼2.45 V vs. Al^3+^/Al^62^. Apart from this, the conductivity of the AlCl_3_–amide systems is rather poor at room temperature (see Table 1)^25,43,74^. The lower conductivity and the sluggish kinetics of the DES electrolytes have been ascribed to their relatively low concentration of active ionic species as well as strong coordination interactions. Atomistic simulations demonstrated that aluminum ions in the RTILs have a weaker coordination and form Al–Cl complexes with a low stability compared to those in some molecular solvents, and this contributes to the facile ion transport and dissociation^75^.

## Corrosion and current collectors

One of the main practical issues to address for the realization of cell prototypes of ADIBs is the high reactivity and corrosivity of aluminum anolytes. For instance, the coin-type cells composed of stainless steel corrode in chloroaluminate melts, requiring the employment of corrosion-free battery cases. Furthermore, contrary to the LIBs, where the Al foil is considered as an established current collector, the current collector for ADIBs at the positive electrode is still under development. Earth-abundant metals, such as aluminum and iron, are easily oxidized in aluminum anolytes at the high voltages of 4.5–5.25 V vs. Li^+^/Li being employed during positive electrode operation^76–78^. Therefore, oxidatively stable conductive materials such as tungsten, molybdenum, and glassy carbon are typically utilized in ADIBs^79,80^. Of note, it has recently been suggested to use titanium nitride (TiN) as a compelling current collector for ADIBs. As reported by Wang et al.^62^, TiN coated on a stainless steel or flexible polyimide substrate can be fabricated by a low-cost and scalable method, such as magnetron sputtering. The TiN oxidative stability in the AlCl_3_:EMIMCl ionic liquid is on par with that of W and Mo current collectors. Notably, graphitic electrodes without a current collector were also proposed by Di-Yan Wang et al.^81^ as an alternative approach to address the oxidation issues. So-called free-standing natural graphite films were fabricated by graphite slurry tape casting on the Cu foil following its etching in an iron chloride (FeCl_3_) solution.

Moreover, the research on the chemical stability of Al foil on the negative side of the AGDIB in acidic RTILs was performed recently by Tak et al.^82,83^. It has been demonstrated that Al surface corrodes in chloroaluminate melts. Furthermore, the corrosion enhances significantly upon increasing their acidity. Importantly, the works of Tak et al.^82,83^, pointed to the fact that additional research efforts should be carried out to mitigate or entirely suppress the reaction of Al with chloroaluminate electrolytes. The quest for non-corrosive electrolyte formulations, therefore, continues.

## Outlook

Although significant advances were accomplished recently on Al electrolytes for ADIBs, substantial room remains for the improvement in their gravimetric/volumetric charge storage capacity and the efficiency of Al plating/stripping. We note that ionic melts used in ADIBs are not just electrolytes (ion-conductors), but represent an electrochemically active, capacity- and rate-limiting battery component. In this context, further research should be focused on finding the practical amounts of the electrolyte needed for the operation of ADIBs. Notably, most of the studies presented in the literature have employed a 2–10-fold excess of the electrolyte required to match the charge storage capacity of the positive electrode (cathode-limited cell). Such tests are acceptable for research purposes, but they do not provide correct and practically relevant information on achievable energy/power densities or the cycling stability of ADIBs. It is also apparent that future research should focus on the decrease in the redox potential of the Al plating/stripping being ca. 1 V higher (~−0.7 V vs. SHE) than that in aqueous solutions (−1.66 V vs. SHE). Additionally, we suggest that subsequent work should stress the other issues associated with ADIB technology, one being the incompatibility of most metallic current collectors with the corrosive Al electrolytes. For instance, aluminum and stainless steel slowly oxidize in the AlCl_3_:EMIMCl ionic liquid when electrochemically polarized up to 2.5 V vs. Al^3+^/Al. Thus far, only tungsten, molybdenum, glassy carbon, chromium, and titanium nitrides have been identified as electrochemically stable current collectors in such batteries. In this framework, we note that any oxidation stability issues in the current collectors that cause a severe decrease in the coulombic efficiency of ADIBs will have a profound effect on their electrochemical performance, which is almost ignored in scientific publications. Towards this end, we state that all these advancements on Al electrolytes should be achieved at a low cost to keep the overall cost-competitiveness of ADIBs.

## Supplementary information

Supplementary Information
