# Supplementary material for: Aluminum electrolytes for Al dual-ion batteries
Source: Commun Chem. 2020 Aug 28;3:120. doi: 10.1038/s42004-020-00365-2 (PMC9814210; doi:10.1038/s42004-020-00365-2)
Supplement: Supplementary file 1 — Supplementary Information [file 42004_2020_365_MOESM1_ESM.pdf]

Supporting Information for

Aluminum electrolytes for Al dual-ion batteries

Kostiantyn V. Kravchyk,[1,2*] *and Maksym V. Kovalenko,*[1,2*]

[1] Laboratory for Thin Films and Photovoltaics, Empa – Swiss Federal Laboratories for Materials Science and Technology, Überlandstrasse 129, CH-8600 Dübendorf, Switzerland

[2] Laboratory of Inorganic Chemistry, Department of Chemistry and Applied Biosciences, ETH Zürich, Vladimir-Prelog-Weg 1, CH-8093 Zürich, Switzerland

Corresponding Author

*E-mails: kravchyk@inorg.chem.ethz.ch and mvkovalenko@ethz.ch

Supplementary Note 1

Charge storage capacity of deep eutectic solvents

The capacity of the deep eutectic solvent anolytes can be calculated as follows (considering that the electrodeposition of Al occurs only in acidic media (r > 1, in the presence of $Al_2Cl_7^-$ ions)):

Gravimetric $C_{an} = \frac{Fx}{m}$ (mAh g^{-1}) (1)

Volumetric $C_{an} = \frac{Fx}{V}$ (mAh ml^{-1}) (2)

where $F = 26.8 \times 10^3$ mAh mol^{-1} (Faraday constant), $x = \frac{3}{4}$ (number of electrons used to reduce 1 mol of the $Al_2Cl_7^-$ ions as follows from the equation: $4Al_2Cl_7^- + 3e^- \leftrightarrow 7AlCl_4^- + Al$) and m and V are the mass (in g) and volume (in ml) of anolyte containing 1 mol of the $Al_2Cl_7^-$ ions.

The mass and volume of an anolyte containing 1 mol $Al_2Cl_7^-$ ions can be calculated as follows:

$m = \frac{\rho}{M} \times 10^3$ (g) (3)

$V = \frac{1}{M} \times 10^3$ (ml) (4)

where M is molarity of $Al_2Cl_7^-$ ions in the anolyte (mol L^{-1}) and ρ is density of the anolyte (g mL^{-1}).

Substituting Eq. 3 and Eq. 4 into Eq. 1 and Eq. 2, one obtains:

Gravimetric $C_{an} = \frac{FxM}{\rho \times 10^3}$ (mAh g^{-1}) (5)

Volumetric $C_{an} = \frac{FxM}{10^3}$ (mAh ml^{-1}) (6)
